# Techno-economic analysis of wind power integrated with both compressed air energy storage (CAES) and biomass gasification energy storage (BGES) for power generation

**DOI:** 10.1039/c8ra03128b

**Published:** 2018-06-14

**Authors:** Chidiebere Diyoke, Mathew Aneke, Meihong Wang, Chunfei Wu

**Affiliations:** School of Engineering, University of Hull Hull UK HU6 7RX c.wu@hull.ac.uk +44(0)1482466464; School of Chemical and Biological Engineering, University of Sheffield Sheffield UK S10 2TN; School of Energy & Environmental Engineering, Hebei University of Technology Tianjin China

## Abstract

A techno-economic analysis of excess wind electricity powered adiabatic compressed air energy storage (A-CAES) and biomass gasification energy storage (BGES) for electricity generation is implemented to determine the performance of the system and the potential profitability of developing such a facility for distributed power generation in the UK by an investor, given the customer's demand for heat and electricity. The customers are considered to be about 1600 households in the Humber region, UK, who use heat generally for space heating and domestic hot water applications. The system is modelled using a developed Matlab computer code and its performance evaluated using total system efficiency (TSE), net present value (NPV) and cost of electricity (COE) as metrics. TSE of 36.8% is obtained for the system while the COE is found to be about £0.19 per kW h. In terms of profitability, the system returned a negative NPV of £2 144 062 signalling the non-profitability of the system in the proposed location. However, if 70% of total investment cost (TIC) of the system is provided for by means of a subsidy, the system becomes economically viable with positive NPV of £132 475 and COE of £0.10 per kW h respectively. The sensitivity study shows that the most significant factors swaying the NPV of the A-CAES-BMGES are TIC, O&M cost, excess wind electricity cost, electricity tariff and cost of diesel fuel.

## Abstract 2

Wind power was integrated with both compressed air energy storage and biomass gasification energy storage for power generation.

## Graphical abstract

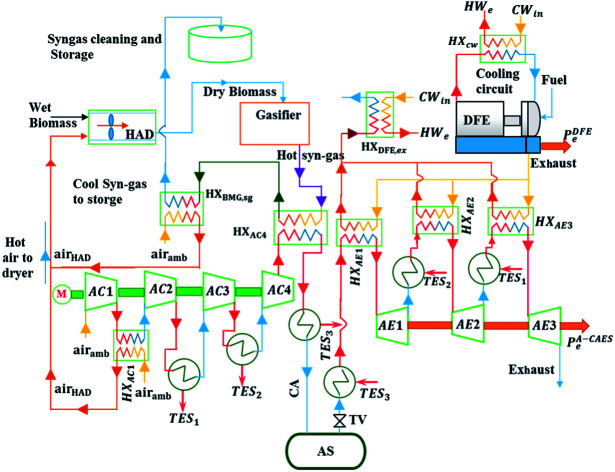

## 1. Introduction

Global energy use is dominated by fossil fuelled sources.^1,2^ In the recent past, the issues of climate change, depleting nature of fossil fuels and the yearly rise in global energy demand has led to a new global energy policy focusing on improvement of energy efficiency, promotion of energy conservation and an energy mix involving increased deployment of renewable energy (RE) sources^1–3^

Wind power generation is one of the RE sources that is continuously becoming attractive for meeting current and future energy needs.^1,2^ This is due to its established technology, wide availability, good scalability and relatively low cost with projected cost of electricity (COE) below 34 $ per MW h for onshore wind compared to 54 $ per MW h for large, ground-mounted PV in the United States at 3% discount factor.^4^ In 2016, wind power accounted for more than half of 14.1% global renewable energy use growth.^5^ It has a significant role to play in the quest to decarbonise the globe and switch from fossil based generation to renewable based generation. Wind is forecasted to have a good significant future cost reduction potential as its technology improves.^4^

However, wind suffer from a major limitation. It is unpredictable and can only provide energy intermittently. The intermittency leads to a mismatch between electricity demand and supply which consequently introduces an enormous challenge of safety and reliability to the grid operation. In addition, the energy density of wind is low in comparison to that found in conventional fossil fuel sources like coal, oil, gasoline and natural gas.^6^

Energy storage (ES) has been identified as a major solution to the intermittency of wind. This is because of its favourable charging and discharging features, which makes it possible to overcome the fluctuations.^7^ ES has the capacity to even out the mismatch between energy supply and energy demand, thus playing a critical role in energy conservation. It also improves the efficiency and reliability of energy system. The increased efficiency would result to energy conservation and increased cost effectiveness.

Different energy storage (ES) technologies exist: Compressed Air Energy Storage (CAES), Pumped Hydro Storage (PHS), Battery Energy Storage (BES), Super Magnetic Energy Storage (SMES) and Flywheel Energy Storage (FES).^1,4,8–15^ Though each of the ES technologies offer exclusive advantages, they also suffer from special disadvantages. CAES is one of the mature and economically attractive ES technologies with capacity to enable a more efficient and flexible energy system with a better use of the fluctuating wind and other renewable energy sources.^3,16^

In a CAES process, during the charge time at low-cost off-peak periods, ambient air is compressed and stored in large underground caverns or above ground storage vessels using excess electricity. During the discharge time at peak period, the stored compressed air is preheated and expanded through a turbine to produce electricity.^17^

Two basic types of CAES system exist: diabatic CAES (D-CAES) and adiabatic CAES (A-CAES).^17^ The D-CAES involves the combustion of fossil fuels with the compressed air (CA) to raise the temperature of the compressed air before expansion *via* gas turbines, leading to CO_2_ emissions. The A-CAES stores the heat released during the compression process and reuse it to heat up the stored compressed air in the expansion process.^17^

An alternative CAES system exists called the Isothermal CAES (I-CAES). Isothermal CAES (I-CAES) technology aims to address some of the diabatic and adiabatic CAES, by eradicating the need for fuel and high temperature heat energy storage, thus, offering an enhanced round trip efficiency in the range of 70–80%.^18–20^ Being an isothermal process, the air is compressed without a change in temperature, thus reducing the work of compression while maximizing the work needed for expansion, through effective heat transfer with surroundings of the air vessel. An experimental study by Alami *et al.*^21^ on low pressure, modular small scale compressed air energy storage (CAES) system for wind energy storage applications working on the I-CAEs principle found that an I-CAES CAES working with a series of off the shelf low pressure cylinders instead of pressurised containers or cavern could be preferable on small-medium scale (1–10 MW),^21^ and has the following advantages: enables the use cheaper, low pressure storage containers, and practically eliminates the need for heat removal considerations necessary in higher pressure systems to offset the temperature rise after compression. Also, it allows close control of discharge rates according to power consumption needs while maintaining minimal losses. The authors also reported a maximum overall system efficiency of about 97.6% for the system, while its physical footprint is less than 0.6 m^3^.

However, while there is substantial research into near-isothermal compression for CAES (by companies like Lightsail and SustainX),^22^ I-CAES is not yet commercially available and any currently available compression that approaches reversible isothermal compression is too slow for industrial use^20,23^ due to the impractically small temperature differences required.^20^ Consequently most reported A-CAES designs opt for a series of adiabatic or poly-tropic compressions, after each of which the compressed air is cooled back to the ambient temperature in order to reduce the both the temperature and volume of the air.^22,24^

There are different options for storing the thermal energy in a CAES process: sensible heat storage (SHS) in liquids and solids, latent heat storage (LHS) in phase change materials (PCMs) and thermochemical heat storage (THS) in chemical reactions.^17^

Although ES can help solve the problem of variability of wind, it does not solve all the problems. Shortfalls resulting from losses in low roundtrip efficiencies of an ES system can be compensated by designing a hybrid ES systems involving the RE source with ES and other energy sources of generation that can be mobilised on short notice and whose generation features must complement each other.^25^ For example, a combination of A-CAES and biomass gasification electrical storage (BMGES) system for meeting the power and heat requirements of a given community. In fact, wind powered A-CAES and BMGES system are complementary to some extent, since some of the heat from syngas produced during gasification can be used by the A-CAES process whereas the biomass gasifier can use some of the cheap heat generated by the compression process of the A-CAES to dry its fuel before gasification. Moreover, combination of A-CAES and BMGES may act as a CHP system, by recovering heat from the dual fuel engine (DFE) during and after the power generation phase. This can only be achieved by placing the A-CAES and BMGES close to the area of energy demand, given the technical difficulty to transfer thermal energy at large distances.^26^ Such a plant allows to maximize and conjugate the extensive benefits of distributed generation (DG)^11^ with those of electricity storage.

Many hybrid energy storage systems involving A-CAES has been proposed and analysed in recent years: Garrison and Webber^27^ proposed a novel hybrid configuration involving solar energy and excess wind electricity powered CAES system in which the air from the cavern is preheated by solar energy rather than natural gas prior to expansion in the turbine. They reported an overall efficiency of 46% for the coupled solar-CAES system. Zhang *et al.*^13^ analysed a hybrid diesel DG system integrated with A-CAES and thermal energy storage (TES). Their results suggested that the hybrid system's exergy efficiency is 41.5%, and the primary fuel saving ratio is 23.13%. In another study by Singh and Baredar,^28^ a techno-economic assessment of a solar PV, fuel cell, and biomass gasifier hybrid energy system was reported. The cost of electricity (COE) and net present cost of the system was reported to be 0.23 $ per kW h (15.064 Rs per kW h) and $79 858.76 (Rs. 5 189 003) respectively.

A biomass-fired combined cooling, heating and power system with TES system was studied by Caliano and Bianco.^29^ Their result suggested that the combined use of a TES and cold TES during the hot season could represent a viable economical solution.

In another related study, Singh *et al.*^30^ carried out optimal sizing and feasibility study of an island micro grid in rural area consisting of PV, wind, biomass and BES system using artificial bee colony (ABC) algorithm. They reported the sizes of the system components as 250 kW solar PV, 19 kW wind turbines, 1400 batteries and 40 kW biomass gasifier.^30^ To verify the strength of the ABC technique, the results obtained were compared with that obtained from the standard software tool, hybrid optimization model for electric renewable (HOMER) and particle swarm optimization (PSO) algorithm. It was reported that ABC provides an optimal configuration with least levelised COE of 0.173 $ per kW h.

A hybrid solar-biomass power plant without ES was studied by Srinivas and Reddy.^31^ Their result indicated that the plant fuel energy efficiency increases from 16% to 29% with an increase in solar participation from 10% to 50% at the boiler pressure of 20 bar.

To provide a solution for managing excess heat production in tri-generation plant and thus increase the power plant annual efficiency, a hybrid optimization model of biomass tri-generation system combined with TES was studied by Dominković *et al.*^32^ Three case studies with minimum yearly average power plant efficiencies of 50%, 65% and 75% respectively were conducted. It was reported that an increase in overall power plant efficiency from 50% to 65% in legislation, in order to be eligible for the maximal feed-in tariff, would not affect the economics of the system.

Hence, from the literatures reviewed so far and within the limits of the author's knowledge, no treatment exists in the literature currently that focused on the technical and economic analysis of wind power integrated with both Compressed Air Energy Storage (CAES) and Biomass Gasification Energy Storage (BGES) System for Power generation in the UK. Downdraft gasifier is suited for small and medium-sized applications.^33^ This conversion technology has a fairly efficient biomass to gaseous fuel conversion and produce syngas with relatively low amounts of tar that is suitable for direct use in internal combustion engines.^33,34^ Hence, a fixed bed downdraft gasifier is selected for this work. Given the advantage of A-CAES system and the need to increase RE in the energy mix around the globe, it could be expected that the combination of wind powered A-CAES and BMGES hybrid system will be an important trend. Hence, in order to investigate this concept, we developed a mathematical model of A-CAES integrated with BMGES system in Matlab. The A-CAES is powered using off-peak electricity from wind turbine. Technical and economic analysis of the hybrid system is analysed using efficiency, Net Present Value (NPV) and Cost of Electricity (COE) as metrics. The impacts of some technical and economic factors on system efficiency and profitability of the of hybrid system are subsequently investigated.

## 2. System description

**Fig. 1:**
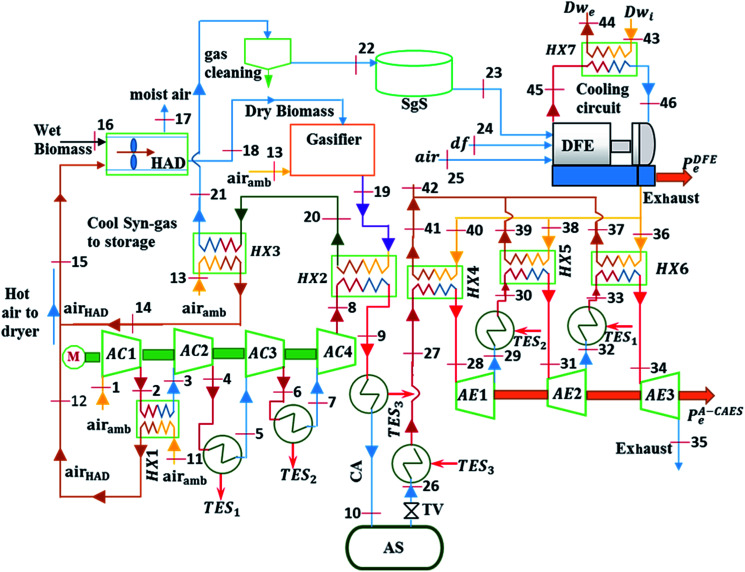
Schematic diagram of the proposed A-CAES + BMGES system.

The system being proposed is as shown in Fig. 1. It is made of the following components: A-CAES powered by excess wind electricity, Hot air dryer (HAD) for drying biomass; Biomass gasifier (BMG) for generating syngas from dried wood, dual fuel engine (DFE) running on syngas and diesel fuel and heat exchangers (HXs).

First the excess electricity of the wind turbine powers the compression train to compress air to a higher temperature and pressure. The heat of compression of the first stage air compressor (AC1) is recovered by the air compressor heat exchanger (HX1) for biomass drying in the HAD, while the heat of compression of the remaining three stages (AC2, AC3, AC4) is recovered and stored as TES_1_, TES_2_ and TES_3_ respectively using the phase changes materials (PCM) in TES tanks, TEST_1_, TEST_2_ and TEST_3_ respectively. The cooled compressed air (CA) is stored in the air store (AS).

Subsequently, wood chips at initial moisture content (MC_i_) of about 50% wt. are dried in the HAD to final moisture content (MC_f_) of about 10% wt. The dried wood is converted into syngas (sg) in the BMG. The heat carried by the generated syngas in recovered by means of the heat exchanger (HX2) and used to pre heat the temperature of the air exiting the last stage air compressor (AC4). The syngas is further cooled by recovering its heat using a heat exchanger (HX3). The recovered heat is used for biomass drying. Cooling the syngas helps to prevent pre-ignition, to improve the engine volumetric efficiency, and to ease gas clean-up.^33^ Afterwards, the cooled syngas which is limited at a temperature of 60 °C, to minimise condensation, is cleaned and stored in the syngas store (SgS). Syngas from a gasifier contain various contaminants and thus has to undergo several cleaning steps to reduce the concentrations of contaminants to level required by the downstream processes. For use in internal combustion engine, typical syngas cleaning requirements is particulate (<50 mg m^−3^), tars (<100 mg m^−3^).^35^ Therefore, wet syngas cleaning, was used in this study. In such a scheme, the syngas is first passed through a cyclone to reduce particulates. The syngas is then further cooled to a temperature below 100 °C and passed through a spray scrubber to remove tar and ranges of other contaminants.^36^

When electricity is required, the stored syngas is used to generate electricity in the DFE while the stored cool CA at high pressure exiting the AS is first throttled down to the minimum operating pressure of the AS (40 bar) using a throttle valve (TV) and then heated using *Q*_TES3_. The high temperature pressurised air leaving *Q*_TES3_ is subsequently heated up further by the exhaust heat from the DFE by means of the heat exchangers (HX4), and then expanded in AE1. After expansion in AE1, the temperature of air exiting the first stage AE (TET_AE1_) is heated up first by *Q*_TES2_ and further again by HX5 before it undergoes expansion in AE2. The process of heating and further heating by *Q*_TES_ and HX is repeated until the last stage AE to produce electricity. Heat is recovered from the cooling water jacket of the DFE using cooling water heat exchanger (HX7).

A techno-economic analysis of the proposed hybrid system has been implemented to determine its value to the utility system and the potential profitability of developing such a facility by an investor, given the customers demand for heat and electricity. The customer is considered to be about 1600 households in the Humber region UK who use heat generally for space heating and domestic hot water applications. For brevity, the proposed hybrid system is hereafter referred to as (A-CAES + BMGES)

### 2.1 Demand profiles

**Fig. 2:**
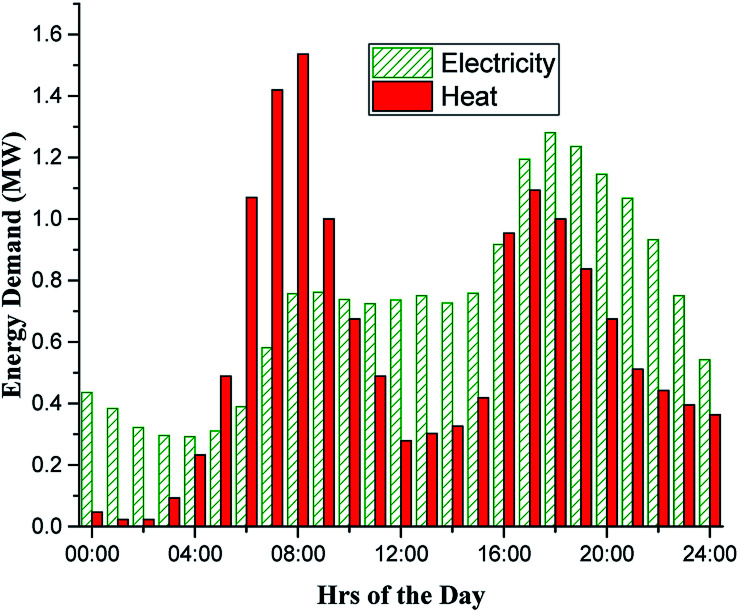
Daily hourly electrical and thermal demand profiles for North Humberside.

To simulate the proposed system performance for the assumed 1600 households, the electrical and heat demand profiles were generated as shown in Fig. 2. The electrical profile was obtained from the data of half-hourly average daily electrical energy consumption in kW h for the period 01/05/2011 to 31/05/2012 for 5554 households from the UK Northern power grid region.^37^ The data was manipulated to give hourly average data based on the average daily electricity consumption of 4115 KW h per household obtained from a DECC report.^38^ The heat demand profiles are based on real hourly average data from a 100 home community housing development in the UK, obtained from a study by Wood.^39^ The data was manipulated to give electricity to heat ratio equal to the national average for UK social housing of 0.28.^39^

## 3. Modelling of the A-CAES + BMGES

The modelling of the proposed system is presented hereunder. To simplify the analysis, the following assumptions is made:

• The system and its sub components in any operating mode attain steady state.^24,40^

• Pressure losses in heat exchangers and pipes is ignored.^24,40^

• There is no change in enthalpy during the throttling process of CA.^24,40^

• Temperature of the CA in the AS is assumed equal to ambient temperature.^24,40^

• Air and the DFE exhaust gas is treated as ideal gas.

• Space heating water enters HX7 at 35 °C and leaves at 55 °C.^12^

• The changes in kinetic and potential energy are neglected.

• Isentropic efficiencies of the compressor and turbine are 0.85 and 0.88 respectively.^13^

• The motor (generator) efficiency of AC (AE) are 0.99 and 0.97 respectively.^13^

• The heat exchangers (HXs) has effectiveness (*ε*) of 0.7.

### 3.1 Plant capacity

The electrical capacity of the proposed system is sized to match the maximum electricity demand of 1300 MW. Any excess/deficit electricity/heat is sold/bought to/from the grid. The DFE is sized to supply 25% of the total power while the A-CAES system supplies the rest.

### 3.2 Gasifier and dual fuel engine model

The design requirement is to generate a rated output (*P*_e,DFE_) in the dual fuel mode using syngas mixed with diesel. The performance of the dual-fuelled engine is characterised by a factor called syngas fraction (sf). It indicates the amount of diesel fuel (df) input that is replaced by the syngas (sg) in the dual fuel mode. Diesel replacement up to 80% is possible.^33^ The thermal power input of the syngas (*P*_th,sg_) and diesel (*P*_th,df_) to the dual-fuel engine is determined as follows:1
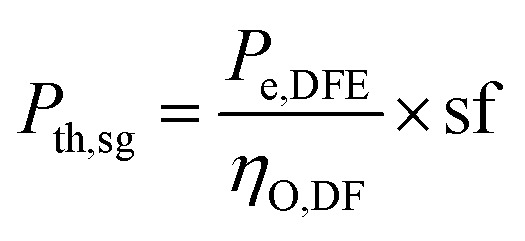
2
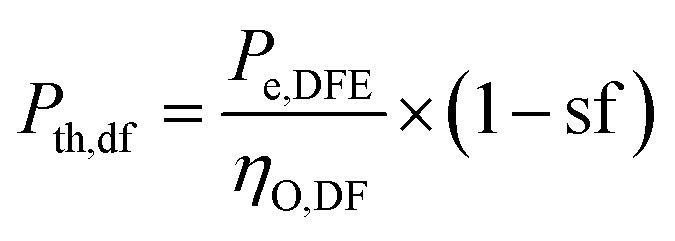


The expected feed rate of biomass on dry basis (d.b) to achieve *P*_th,sg_ is calculated as follows:3
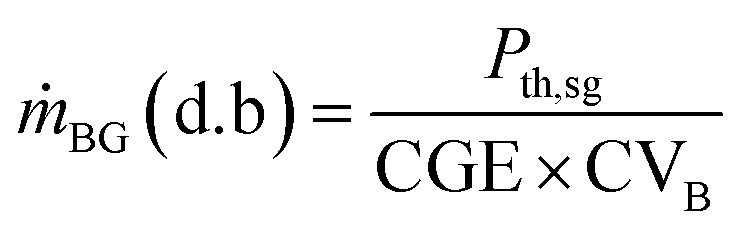


### 3.3 Dryer

**Fig. 3:**
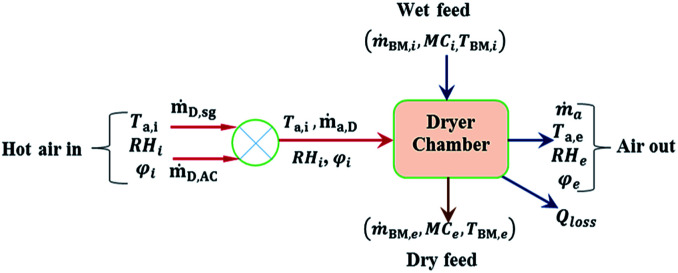
Hot air dryer system.

According to the HAD, as in Fig. 3, the mass balances in the dryer (D) can be written for the dry biomass (B), air (a) and water (w) as follows:

4 *ṁ*_B,D,i_ = *ṁ*_B,D,e_

5 *ṁ*_a,D,i_ = *ṁ*_a,D,e_

6 *ṁ*_a,D_(*φ*_i_ − *φ*_e_) = *ṁ*_w,ev_ = *ṁ*_w,i_ − *ṁ*_w,e_

where *ṁ* is mass flow rate, *φ* is the humidity ratio and subscripts a, D, B, i, e, w and ev denotes air, dryer, biomass, in, exit/final, water and evaporation for specie involved respectively. The mass flow rate of air entering the HAD (*ṁ*_a,D_) is obtained as follows:

7 *ṁ*_a,D_ = *ṁ*_D,AC_ + *ṁ*_D,sg_

where *ṁ*_D,AC_ and *ṁ*_D,sg_ denote mass flow rate of air recovered from AC1 and HX3 respectively. The initial feed rate of wt wood chips to the dryer (*ṁ*_B,i_) at initial moisture content (MC_i_) on wet basis is derived from the feed rate of wood in the biomass gasifier (*ṁ*_BG_) as follows:8
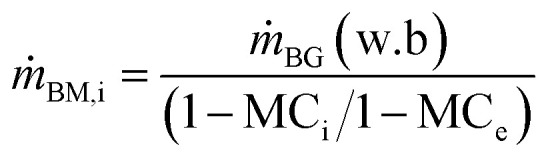


The heat required for drying (*Q*_D_) was obtained from energy balance as follows:^41,42^

9 *Q*_D_ = *Q*_ev_ + *Q*_s_ + *Q*_l,D_

*Q*_ev_ is the heat required to evaporate water, *Q*_l,D_ is heat loss from dryer structures and *Q*_s_ is sensible heat required to heat the biomass and drying structure to drying temperature. where:

10 *Q*_ev_ = *ṁ*_W,ev_[*C*_p,w_(*T*_ev_ − *T*_ref_) + *C*_p,wv_(*T*_a0_ − *T*_ev_) + *L*_wv_]

11 *Q*_s_ = *Q*_B,i_ − *Q*_B,e_

12 *Q*_B,i_ = [*ṁ*_W,i_*C*_p,w_ + *ṁ*_B,e_*C*_p,B_](*T*_B,i_ − *T*_ref_)

13 *Q*_B,e_ = [*ṁ*_W,e_*C*_p,w_ + *ṁ*_B,e_*C*_p,B_](*T*_B,e_ − *T*_ref_)

Heat loss (*Q*_l,D_) is expressed as 5% of the heat input (*Q*_D_). The heat input to the dryer, *Q*_D_ is supplied by the hot air as it is cooled from its initial state (*ṁ*_a,D_, *T*_a,i_, RH_i_) to final state (*ṁ*_a,D_, *T*_a,e_, RH_e_). Thus the energy supplied by the hot air (HA) is as follows:

14 *Q*_HA_ = *ṁ*_a,D_(*h*_a,i_ − *h*_a,e_)

where

15 *h*_a_ = [*C*_p,a_ + *φC*_p,w_](*T*_a_ − *T*_ref_)

In most hot air dryers, the exit temperature of the dried solid is a few degrees lower than the final temperature of the hot air.^43^ In this model, the difference between *T*_a,e_ and *T*_B,e_ is set to be 5 °C.

With known *ṁ*_a,D_ determined from eqn (7), eqn (6) and (9) are solved to obtain the final humidity of air at the exit of the HAD and the temperature of the exiting air and biomass respectively.

The fan electrical power required for circulation of air in the dryer is calculated as follows:

16 *P*_e,fan_ = *ψQ̇*_air_

*ψ* is the specific fan power. In this model *ψ* is set to 1.5 kW m^−3^ s^−1^ as found by Nilsson.^44^

### 3.4 Dual fuel engine (DFE) exhaust flow

From the known fuel input energy, the flow rate of the syngas and diesel fuel is calculated as follows:17
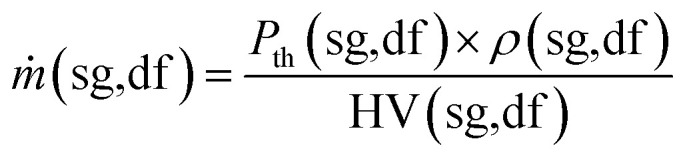


The exhaust gas flow rate of the DFE is estimated using:

18 *ṁ*_ex_ = *ṁ*_df_ + *ṁ*_sg_ + 0.5*Nη*_v_*V*_s_*ρ*_a_/60

Table 1 shows the HAD and DFE input design parameters. The relevant characteristic parameters of the selected diesel engine has been take from the manufacturer's specification.^45^

**Table 1:** Dryer and DFE Design parameters

Parameters	Value	Ref.
**Dryer**		
Feed rate of wet wood, *ṁ*_w,BM,in_ (kg h^−1^)	636.1	
Initial moisture content of feed MC_i_ (%)	50	
Exit moisture content of feed MC_e_ (%)	10	
Temperature of the wood feed, *T*_BM,i_ (°C)	20	
Temperature of the air feed, *T*_a,i_ (°C)	80	
Humidity of air feed, *φ* (kg per kg dry air)	0.006	
Specific heat capacity of dry air, *C*_p,a_ (kJ kg^−1^ K^−1^)	1.006	
Specific heat of water, *C*_p,wv_ (kJ kg^−1^ °C^−1^)	4.186	
Latent heat of water vapour LH_w,v_ (kJ kg^−1^)	2256	
Specific heat of dry feed *C*_p,BM_ (kJ kg^−1^ K^−1^)	1.236	
Specific heat of water vapour, *C*_p,wv_ (kJ kg^−1^ K^−1^)	2.01	

**DFE**		
Rated power output, *P*_e,DFE_ (MW)	0.3	
Exhaust temperature, *T*_ex_ (°C)	461	45
Overall efficiency in dual fuel mode *η*_O,DF_ (%)	20	46
Calorific value of syngas, CV_sg_ (MJ m^−3^)	5.135	
Calorific value of diesel, CV_df_ (MJ kg^−1^)	45.5	47
Engine speed, *N* (rpm)	1000	45
Engine capacity, *V*_s_ (l)	91.6	45
Volumetric efficiency, *η*_v_ (%)	80	33

### 3.5 A-CAES system

#### 3.5.1 Compressor

Assuming maximum work per compressor stage, the mass flow rate of air in each compressor stage (*ṁ*AC) is calculated as follows:19
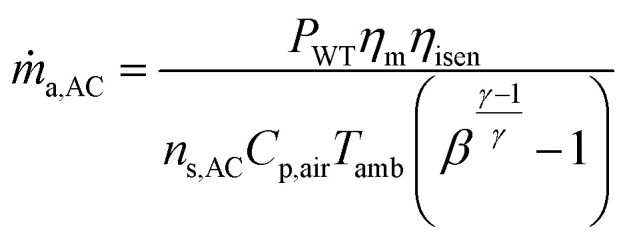


The pressure ratio of each compressor (*β*_AC_) stage is a function of the number of stages (*n*_s,AC_) and overall pressure ratio (*β*_O,AC_) of the compressor. It is calculated using the following equation:20
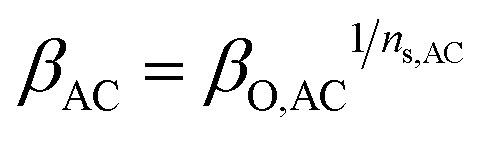


The outlet temperature from the compressor is obtained using the relation:21
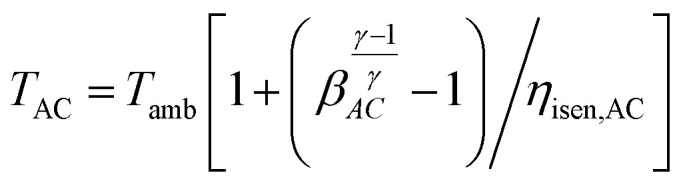


The heat recovered from AC1 for biomass drying (RH_AC1_) was obtained as follows:

22 RH_AC1_ = *η*_HX_*ṁ*_a,AC_[*h*_e_ − *h*_i_]

The mass flow rate of air from AC1 for biomass drying is obtained as follows:23
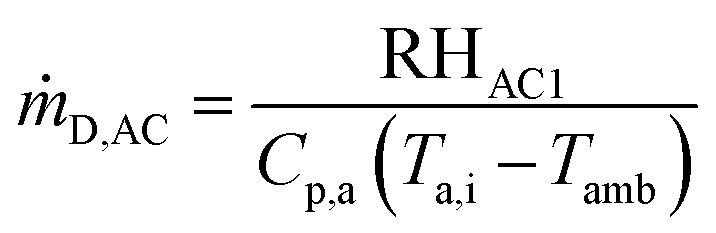


The syngas (sg) produced from a downdraft BMG is drawn from the high temperature reduction zone operating at about 815–1200 °C.^48^ At the point of leaving the gasifier, the temperature of the hot syngas (*T*_sg,i_) lies in the range of about 500–816 °C.^48,49^ For a given heat exchanger effectiveness (*η*_recup_), the air temperature leaving the Cc HX2 and entering the TEST is obtained as follows:24
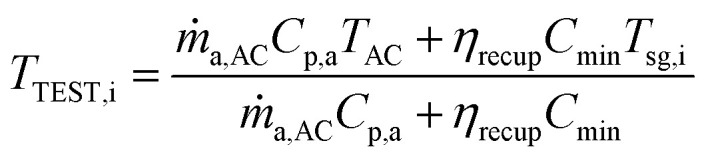
where *C*_min_ is min [*ṁ*_a,AC_*C*_p,a_, *ṁ*_sg_, *C*_p,sg_]

The temperature of the syngas leaving the Cc HX (*T*_sg,O_) is obtained by energy balance. As the temperature of the air exiting the compressor is somewhat high, all the heat contained in the syngas from the gasifier is not fully recovered. If the temperature of the syngas exiting the HX2 is more than 200 °C, the heat it contains is recovered further using syngas cooler HX3 for use in generating hot air at a temperature of about 80 °C for biomass drying while the syngas is cooled to a final temperature not less than 60 °C

The heat recovered from syngas cooler HX3 for biomass drying (RH_D,sg_) was expressed as follows:

25 RH_sg_ = *η*_HX_*ṁ*_a,sg_[*h*_e_ − *h*_i_]

The mass flow rate of air from HX3 for biomass drying is obtained as follows:26
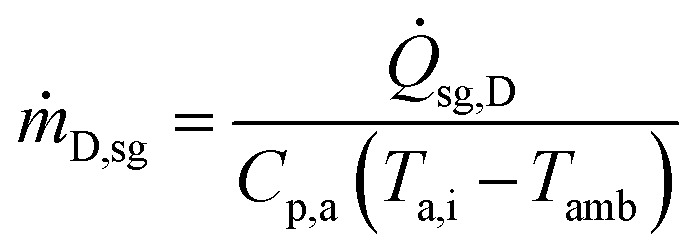


#### 3.5.2 Thermal energy storage tank (TEST)

The thermal storage capacity of the TEST is expressed as follows:

27 *Q*_TES_ = *ṁ*_a,AC_*C*_P,air_*η*_TES_[*T*_TEST,i_ − *T*_PCM,c,_]

PCM thermal storage media has been considered in the present analysis because among the various heat storage techniques, latent heat (LH) TES has the capacity to provide large heat storage capacity and isothermal behaviour during the charging and discharging processes. The other benefits of LH TES include compactness in sizes and low weight per unit storage capacity.^50^ To achieve a suitable TES, the highest melting point of the PCM in each TEST must match the required turbine inlet temperature for each stage (TIT_sn_). The chosen melting point of PCM (*T*_m_) in each TEST is related to the entering temperature of the air (*T*_TEST,in_) according to the relation *T*_m_ = *T*_TEST,i_ − 10 °C. during the discharge mode, the temperature of the PCM when cold (*T*_PCM,c_) must always be above the temperature of the cold pressurised air entering the TEST. In the model, *T*_PCM,c_ is set as 50 °C, 70 °C and 50 °C for TEST_1_, TEST_2_ and TEST_3_ respectively. Table 2 shows the thermo physical properties and cost of the PCMs.

**Table 2:** Thermo physical properties of the PCMs.^54^

TEST	Eutectic compounds	Mass ratio	*T*_m_ °C	*L* kJ kg^−1^	*C*_p,sol_, KJ kg^−1^ K^−1^	*C*_p,liq_, KJ kg^−1^ K^−1^	*ρ*_sol_, kg m^−3^	Price	
								£ per m^3^	£ per kW h
1 and 2	KNO_3_–NaNO_2_	56–44	141	97	1.18	1.74	1994	504	9.7
3	LiNO_3_–NaNO_3_	49–51	194	262	1.35	1.72	2317	3084	19

The mass (*m*) of the PCM in each TEST is estimated as follows:^51^28
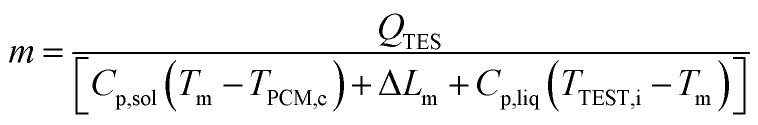
where subscript *m* stands melting, sol for solid and liq for liquid. The magnitude of the recovered energy depends on the roundtrip efficiency (RTE) of the charge/discharge cycles. The outlet temperature of the air from the TEST (*T*_TEST,O_) is calculated thus:29
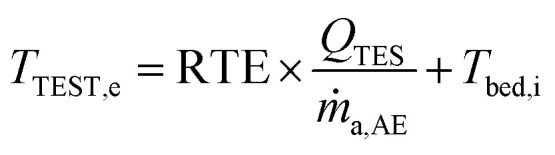


The temperature of the air leaving HX4, HX5 and HX6 and entering the AE1, AE2 and AE3 respectively (TIT) in each stage is obtained by energy balance as follows:

30 TIT_AE_ = *T*_TEST,e_ + *ṁ*_ex_*C*_P,ex_(*T*_ex,in_ − *T*_ex,O_)/*ṁ*_AE_*C*_P,air_

The outlet temperature of the DFE exhaust gas (*T*_ex,O_) is set to the stack temperature of the selected dual fuel engine which is 120 °C to prevent the corrosive effects of condensation in the exhaust piping.^52^

#### 3.5.3 Air store volume

The output power of the A-CAES system depends on the volume of air compressed during charging. The volume of the storage chamber (*V*_AS_) is estimated as follows:31
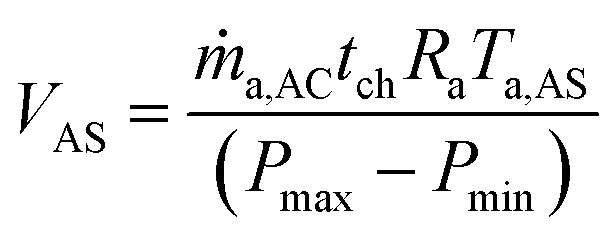


#### 3.5.4 Air expander (AE)

The outlet temperature of air exiting the AE in each stage *T*_AE,o_ is calculated as follows:32
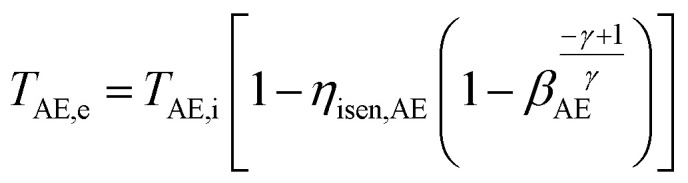


The charge and discharge time ratio the AC to the AE is directly dependent on the discharge to charge mass ratio and its density and can be represented as:^53^33
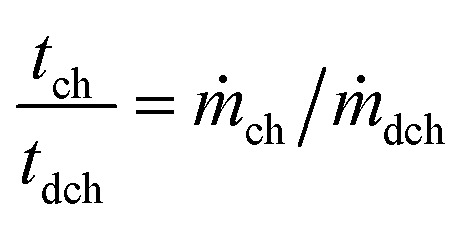


The generated power output by the A-CAES (*P*_AE_) and the total power output by the hybrid system (*P*_T_) at a given instant is calculated by the following equations:34
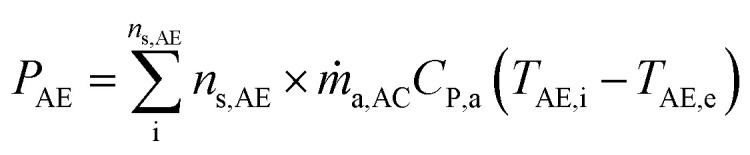

35 *P*_T_ = *P*_AE_ + *P*_e,DFE_

#### 3.5.5 Waste heat recovery (WH) for district heating (DH)

In the present analysis, the supply of medium low pressure hot water at a network supply temperature of 55 °C for domestic hot water or district heating (DH)/low grade heat process requirements^55,56^ is considered. Due to the low temperatures, Counter current (Cc) shell-and-tube type heat exchangers with high efficiency and low operating temperature difference is considered.^57^ Heat can be recovered from three sources in the system (if available). These are the AE exhaust, the cooling water jacket and the hot exhaust gas.^58,59^ In typical engines, 30% of the fuel energy input is contained in the jacket water and is capable of producing 90 to 99 °C hot water.^52^

Based on the thermodynamic analysis using energy balances, the following equations can be used to estimate the recovered thermal energy from the cooling water jacket. The heat content of the cooling water is estimated based on the assumption that 30% of the fuel input is lost in the cooling water.^52^

36 RH_cw_ = 0.3*η*_HX_(*P*_th,sg_ + *P*_th,df_)

Counter current flow configuration is considered for all HXs and the area of all heat exchangers is modelled using LMTD method.^60^ The energy balance equations applied in the HX is as follows:

37 RH_cw_ = *ṁ*_c_[*h*_c,e,_ − *h*_c,i_] = *ṁ*_h_[*h*_h,i_ − *h*_h,e_]

where the subscript c and h denote cold and hot respectively. The heat transfer area of the HX is obtained as follows:38
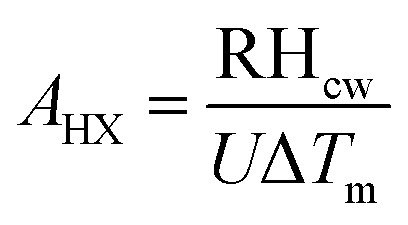


In the analysis, *U* value of 120W m^−2^ K^−1^, is assumed.^61^ Similar procedure is followed for the calculation of the area of other HXs.

### 3.6 System performance criteria

#### 3.6.1 Energy efficiency

The system energy efficiency is analysed with two parameters: the system electrical efficiency (SEE) and the total system efficiency (TSE). SEE is the ratio of the useful net energy output from the system to the total energy input. TSE is the ratio of the sum of the net useful electric output and net useful recovered heat from system, to the total fuel energy input.^40,53,62^

39 SEE = *P*_T_*t*_dch_/(*P*_WT_ + *E*_fuel_)*t*_ch_

40 TSE = (*P*_T_ + RH_cw_)*t*_dch_/(*P*_WT_ + *E*_fuel_)*t*_ch_

where *E*_fuel_ is the total fuel input consisting of biomass energy input and diesel fuel energy inputs to the DFE system

The term useful output (for both electricity and heat) covers all the electricity or heat produced, no matter what the demand in the study area is. Table 3 shows the values for the input parameters used in the A-CAES model.

**Table 3:** Input parameters used in the CAES model

Parameter, symbol (unit)	Value	Ref.
Ambient temperature, *T*_amb_ (°C)	25	
Ambient pressure, *p*_amb_ (bar)	1.0132	
Rated power of compression train, *P*_AC_ (MW)	1	
Compressor overall pressure ratio, *P*_r,AC_	70	63
Ratio of specific heats, *γ*	1.4	
Gas constant, *R* (kJ kg^−1^ K^−1^)	287	
Round trip efficiency of TEST, *η*_RTE_	0.7	
Air store min/max pressure, *P*_min_ (bar)	40/70	63
Charge–discharge mass flow ratio	4/3	
Inlet pressure of AE1, *P*_AE1_ (bar)	40	
Expander overall pressure ratio, *P*_r,AE_	40	63

### 3.7 Equipment cost estimation

The hybrid system ids made up of different components (*x*). The total investment cost of component (*x*) is made up two cost factors:^64^

• Final installed cost or direct capital cost (*c*_*x*_): the purchased equipment costs (*c*_*x*_) and other costs (*c*_o_) associated with equipment installation, piping, instrumentation, controls, electrical equipment.

• Indirect costs (*c*^i^_*x*_): cost of materials, land, civil structural and architectural work, and service facilities.

The total capital cost (*c*^T^_*x*_) is the sum of the final installed cost *c*_*x*_ and indirect costs (*c*^i^_*x*_) *i.e.* c^T^_*x*_ = *c*_*x*_ + *c*_o_+*c*^i^_*x*_.

The cost equation used in the model for the calculation of purchased equipment cost for HXs, AE and AC in (£) is as follows:

41 *c*_HX_ = (2800 + 54*A*^1.2^_HX_) × cf_$–£_ × (1 + *e*)^BY−CY^**(ref. 66)**

42 *c*_AC_ = (400 × cf_$–£_) × (1 + *e*)^BY–CY^**(ref. 66)**

43 *c*_AE_ = (400 × cf_$–£_) × (1 + *e*)^BY–CY^**(ref. 66)**

where is cf_$−£_ conversion factor for dollar to pounds; BY is base year and CY is currency year.

The capital cost of the TEST including the AS is made up of the capital cost of the steel tanks, the PCM filling it and insulation as follows:^67^

44 *c*_TEST_ = (*V*_steel–TEST_ + *V*_steel–AS_)*ρ*_steel_ × *c*_steel_ + *M*_PCM_ × *c*_PCM_ + *A*_insu_ × *c*_insu_

In the model, an insulation thickness of 0.038 m and density of steel of 7900 kg m^−3^ is used.^67^ The cost of stainless steel (*c*_steel_) is obtained as 3.18 £ per kg from.^68^ The capital cost of the A-CAES assembly is estimated with the following equation:

45 *c*_CAES_ = *c*_AC_ + *c*_TEST_ + *c*_AE_

The specific capital cost of biomass gasifier coupled to an internal combustion engine electric generator set (BMG + EGS) for power generation and CHP was quoted by IEA^69^ to lie in the range of 3000 to 4000 ($ per kW_e_) in year 2007. Because of technology learning, the lower value of 3000 $ per kW_e_ is used in the analysis and increased by 5% for contingencies and BOP.

The purchase equipment cost of the hot air dryer (HAD), cyclone and scrubber was obtained by scaling using the “six-tenths rule”.^65^ In this approach, the costs (*c*_G_) of a given plant size (*ṁ*_G_) is obtained from a known or reference plant size (*ṁ*_ref_) and cost (*c*_ref_) using the following equation:^65^46
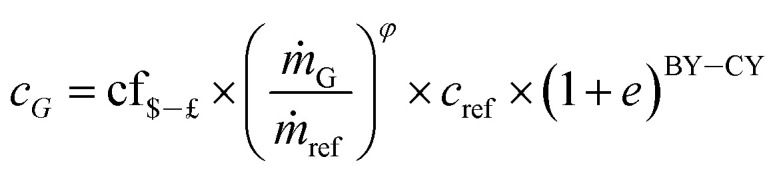
where *φ* is a scaling factor.

Table 4 shows the reference cost factors used in the computation. An average *φ* value of 0.6 has been used in the analysis.^65^ Finally, cost of the hot water tank (*c*_HWT_) vary widely depending on its volume, quality and its capabilities. In the model, a modest low cost value of 925 (£ per m^3^) has been used.^70^

**Table 4:** Reference costs of equipment with their maximum size

Unit	Base capacity	Base cost ($)	CY	Ref.
HAD	500 kg h^−1^	19 891	2010	43
Cyclone	9 m^3^gas per s	1.36 × 10^6^	2002	36
Scrubber	9 m^3^ gas per s	4.56 × 10^6^	2002	36

Cost escalation factor (*e*) also called inflation rate, is used to account for the increase in cost of components and services over time. Naturally, these cost parameters are all subjected to strong uncertainties; therefore, in the present study all item costs were escalated from their base year to 2017 using only one inflation rate *e*.

### 3.8 Economic analysis of the integrated system

To evaluate the proposed integrated system economic performance, the cost of electricity (COE), total life cycle cost (TLCC) and net present value (NPV) were computed. The COE is the minimum price at which energy must be sold for an energy project to break even.

First an estimate of the TLCC for the system is carried out as follows:^71^.

47 TLCC = [TIC − Tax × (*c*_D_)_PV_ + (1 − Tax) × {(*c*_O&M,_)_PV_ + (*c*_En_)_PV_}]

The total investment cost of the integrate system is the sum of the total capital cost (*c*^T^_*x*_) of the various components that make up the system as follows:48
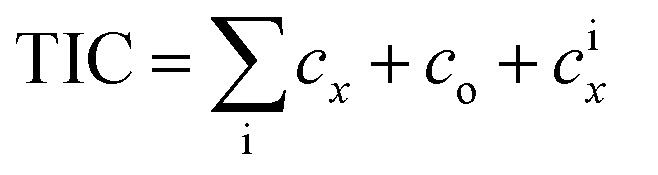


The remaining direct costs (*c*_o_) and indirect costs (*c*^i^_*x*_) are related with equipment installation, piping, instrumentation, controls, electrical equipment and materials, land, civil structural and architectural work, and service facilities. These costs cannot be estimated directly. In the model, they are calculated as 5% of the purchased equipment costs *i.e.* (*c*_0_ + *c*^i^_*x*_ = 0.5_*x*_)

The annual operation and maintenance (O&M) cost can be expressed as a percentage of the capital cost.^4,11^ In the model, the O&M cost is estimated as 5% of the TIC.^4^ The annual cost of the input energy (*c*_En,i_) *i.e.* the cost of biomass (*c*_w,BM_), diesel (*c*_df_) and excess wind energy (*P*_WT_) cost (*c*_r,e_) used by the integrated system is estimated as follows:

49 *c*_En,i_ = hrs_yr_ × {(*Q̇*_df_ × *c*_df_ + *ṁ*_BM_ × *c*_W,BM_) + *P*_WT_ × *c*_r,e_}

50 *c*_W,BM_ = −0.0129MC^2^ − 0.0673MC + 83.925

The cost of wet biomass feed (*c*_w,BM_) at any moisture content (MC) for industry is estimated as follows:

Data for fitting *c*_w,BM_ above has been taken from the report AEBIOM-wood fuels handbook.^72^ Finally the COE is calculated as follows:^71^.51
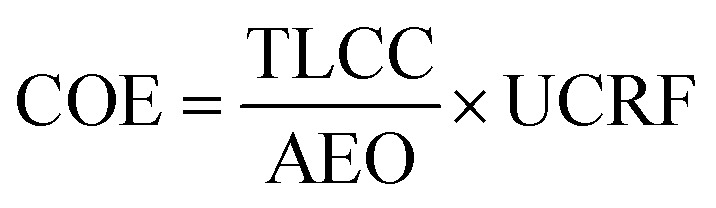


The ultimate capital recovery factor (UCRF) is calculated using.52
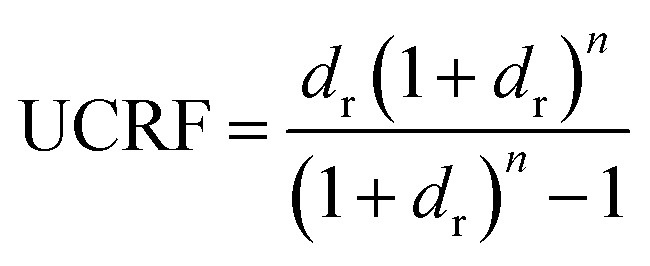


The real discount (*d*_r_) rate is calculated from the nominal discount rate (*r*) using the following formula:^71^.

53 *d*_r_ = [(1 + *r*)/(1 + *e*)] − 1

In the constant dollar analysis, it is necessary to convert cash flows for depreciation from current to constant dollars using the inflation rate (*e*). The following expression shows the conversion from current depreciation (*D*) to constant depreciation (*D*_r_):

54 *D*_r_(*n*) = *D*(*n*) × (1 − *e*)^*n*^

The present value (PV) of the O&M, energy cost (*c*_En_) and depreciation (*c*_D_) is obtained by multiplying the appropriate energy cost, with the present value factor as follows.55
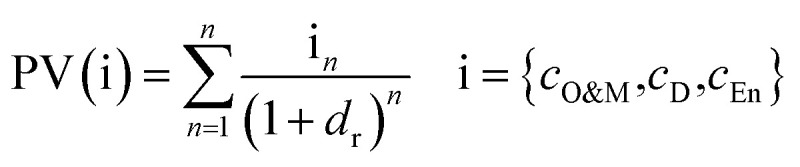


The net present value (NPV) which represents the present value of all the expenses and revenues streams over the life of the system is evaluated with the following:^11,71^56
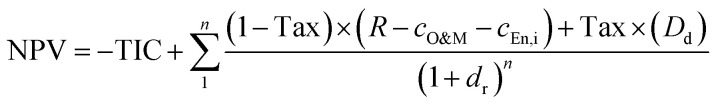
where *R* is the revenue generated by selling electricity and heat produced by the system. *R* is estimated as follows:

57 *R* = *R*_e_ + *R*_h_

58
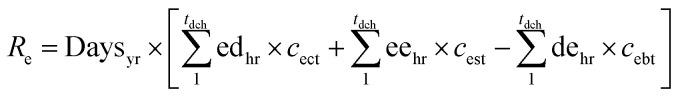
59
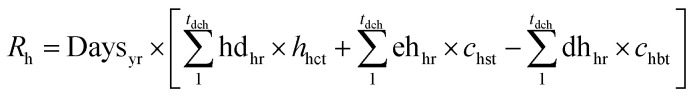


**Fig. 4:**
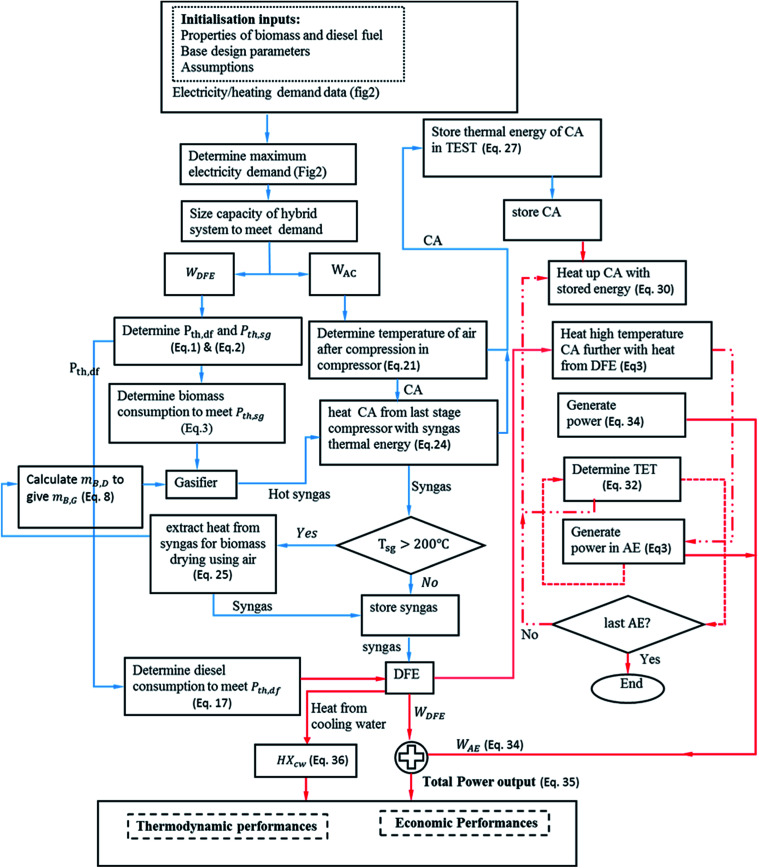
Computational algorithm for the implementation of the model in Matlab.

In the model, the excess wind electricity price (*c*_r,e_) while charging is assumed to be 42 £ MW per h, the 2017 average off-peak spot price.^73^ For discharging (selling), the electricity consumer tariff/price (*c*_ect_) is assumed to be 14.05 p per kW h, the UK national average price per kW h for electricity.^74^ The electricity buy back tariff (*c*_ebt_) and the electricity sell back tariff (*c*_est_) is assumed as 43 £ MW per h, the average 2017 wholesale electricity price in the UK.^75^Table 5 shows the representative values for the main cost parameters of the integrated system. All results have been reported in the 2017 constant dollar year. The methodology described in the preceding section is applied to analyse the performance of the system. Fig. 4 shows a physical set up of the algorithm for the implementation of the model in Matlab.

**Table 5:** Main cost parameters of the integrated system

Parameter, symbol (unit)	Value	Ref.
Diesel fuel cost, *c*_df_ ($ litre^−1^)	1.15	76
Inflation rate, *e* (%)	3	
Rejected wind electricity cost, *c*_r,e_ (£ per kW h)	0.042	
Electricity consumer tariff, *c*_ect_ (£ per kW h)	0.14	
Electricity sell to grid tariff, *c*_est_ (£ per kW h)	0.042	
Electricity buy back tariff, *c*_ebt_ ($ £)	0.042	
Heat consumer tariff, *c*_hct_(£ per kW h)	0.10	77
Heat sell back tariff, *c*_hst_ (£ per kW h)	0.10	77
Heat buy back tariff, *c*_hbt_ (£)	0.10	77
Cost of diesel fuel, *c*_df_ (£ per litre)	1.15	76
Insulation cost, *c*_ins_ ($ m^−2^)	235	68
Nominal discount rate, *d* (%)	10	4
Charging time (discharging time) (h)	8 (6)	
Annual operating days	310	
Economic life, *n* yrs	20	
Tax rate, *T* (%)	34	71

## 4. Results and discussion

Table 6 lists the values of pressure (bar) temperature (K) and mass flow rate (kg s^−1^), determined for the each stream of the system. Using the table data and the equations developed in the preceding section, performance parameters for the system has been calculated. Under design conditions, the HAD is capable of producing 318.03 kg of dry wood per hour with an efficiency of about 53.8% from an initial wet wood feed rate of 636.05 kg per hour. To achieve this, the HAD uses 190.8 kW of thermal energy supplied from recovered thermal energy from AC_1_ (173.25 kW) and syngas (61.98 kW). At 80% syngas fraction (sf), the overall diesel and syngas consumption to deliver an electric output of 300 kW_e_ in dual fuel mode is estimated as 23.74 and 885.24 kg h^−1^ respectively. This corresponds to a biomass and air feed flow rate of 318.03 and 617.65 kg h^−1^ respectively in the BMG, in order for the DFE to deliver 300 kW_e_ in the dual fuel mode.

**Table 6:** Stream properties of the system

No.	*T* (K)	*P* (bar)	*ṁ* (kg s^−1^)	No.	*T* (K)	*P* (bar)	*ṁ* (kg s^−1^)
1	298.0	1.01	1.98	24	298.0	1.01	0.01
2	422.3	2.93	1.98	25	298.0	1.01	0.74
3	298.0	1.01	1.98	26	303.1	40.0	1.98
4	422.3	8.48	1.98	27	403.1	40.0	2.64
5	298.0	1.01	1.98	28	443.1	40.0	2.64
6	422.3	24.5	1.98	29	327.6	11.7	2.64
7	298.0	1.01	1.98	30	383.2	11.7	2.64
8	422.3	70.9	1.98	31	425.8	11.7	2.64
9	491.1	70.9	1.98	32	314.8	3.42	2.64
10	303.1	70.9	1.98	33	376.0	3.42	2.64
11	298.0	1.01	3.13	34	418.6	3.42	2.64
12	353.0	1.01	3.13	35	309.4	1.00	2.64
13	298.0	1.01	1.12	36	734.0	1.01	0.33
14	353.0	1.01	1.12	37	393.0	1.01	0.33
15	353.0	1.01	4.25	38	734.0	1.01	0.33
16	293.0	1.01	0.18	39	393.0	1.01	0.33
17	307.6	1.01	4.25	40	734.0	1.01	0.33
18	302.6	1.01	0.09	41	413.1	1.01	0.33
19	973.0	1.01	0.26	42	399.7	1.01	0.99
20	563.3	1.01	0.26	43	308.0	1.01	3.76
21	377.6	1.01	0.26	44	328.0	1.01	3.76
22	377.6	1.01	0.26	45	363.0	1.01	2.51
23	377.5	1.01	0.25	46	333.0	1.01	2.51

The total electricity produced is 2.18 MW h per year with the DFE contributing about 25.76%, and the A-CAES system about 74.24%. It is interesting to observe that about 86% of the excess wind electricity used during the charging mode is produced in the A-CAES during the discharging mode. This characteristic demonstrates the hybrid nature of the A-CAES + BMGES system and it is so because of the added syngas and exhaust heat energy which is used to further heat up the air to a higher temperature after compression in the compressor and during expansion mode respectively. The total heat recovered from the system for district heating is about 586.37 MW h_th_ per year representing only about 0.16% of the total input fuel energy. This recovered heat, is 100% from the DFE cooling system jacket water.

Although about 60 to 70% of the energy input of the fuel in the DFE is contained in the thermal energy contained in the exhaust gas and cooling systems,^52^ the total percentage of the recovered heat is low because part of the heat content of the exhaust gas is used to heat up the air after the TEST before expansion in the AE stages. The system electrical efficiency is about 28.97%; the total system efficiency for the production of heat and power is about 36.81%. Table 7 shows the technical performance of the overall integrated A-CAES + BMGES system. The thermal storage capacity of the thermal energy storage tanks, TES_1_, TES_2_ and TES_3_ is estimated as 0.19, 0.16 and 0.36 MW respectively. As can be observed, the thermal capacity of TES_3_ is about double that of TES_1_ and TES_2_. This can be explained by higher temperature difference in TEST_3_ made possible by highest and lowest temperature of HTF entering the TEST during the charging and discharging modes respectively when compared with TEST_1_ and TEST_2_.

**Table 7:** Technical performance results for the integrated system

Parameter, symbol (unit)	Case 1
Air compressor power input, *P*_AC_ (MW)	0.99

**Thermal storage capacity of TEST, *Q***_**Th,TEST**_ **(MJ s** ^**−1**^ **):**	
TES_1_	0.19
TES_2_	0.16
TES_3_	0.36
Total electricity output, *P*_T_ (MW_e_)	1.16
System electrical efficiency, SEE (%)	29.0
Total system efficiency, TSE (%)	36.8
Air storage (AS) size, *V*_AS_ (m^3^)	1549

**Heat recovery:**	
Heat recovered from compressed air, RH_AC_ (MW_th_)	0.17
Heat recovered from syngas, RH_sg_ (MW_th_)	0.06
Total heat recovered during charging, RH_t,ch_ (MW_th_)	0.24
Total heat recovered discharging, RH_t,dch_ (MW_th_)	0.32
Heat required by dryer, (kW_th_)	0.20
	
**Area of heat exchangers:**	
*A*_HXAC1_ (m^2^)	147.4
*A*_HX_AC4_ (m^2^)	3.29
*A*_HXBMG,sg_ (m^2^)	0.91
*A*_HXAE1_ (m^2^)	8.50
*A*_HXAE2_ (m^2^)	8.72
*A*_HXAE3_ (m^2^)	7.38
*A*_HX,DFE,ex_ (m^2^)	0
*A*_HX,DFE,cw_ (m^2^)	47.11

The analysed A-CAES + BMGES system is not a single ES technology but a hybrid ES system. Thus comparing its efficiency with an A-CAES system can be misleading. However, the efficiency values computed (both SEE and TSE) assume fairly moderate values when compared with data found in the technical literature for both A-CAES and similar hybrid systems. As a matter of fact, Garrison and Webber^27^ reported an overall efficiency of 46% for a coupled solar-CAES system. Zhao *et al.*^78^ reported RTE ranging from 70–74 for a hybrid A-CAES and flywheel energy storage system (FESS). In another study, electrical efficiencies ranging from 24.79–62.09 was reported by Karellas and Tzouganatos^79^ for an A-CAES system of different configuration (*i.e.* different stages with and without air preheating). Liu and Wang^24^ presented RTEs that vary between 53.9% and 66.98%.

Facci *et al.*^26^ estimated system electrical efficiencies of 30.2% for tri-generative compressed air storage (T-CAES) compatible for a small to medium size civil application. It was reported that the efficiency could vary between 20 to 40% depending on the operating storage pressure (10–100 bar).^80^ In another study Kim and Favrat^81^ presented efficiencies that vary from 23.6% to 71.6%. The wide variation in the reported efficiencies of CAES emanates from the fact that different configurations, operating parameters and conditions are applied by the various authors in their analysis. Such parameters include (a) the average storage pressure, (b) the compressors and turbines efficiencies, (c) the number of stages of AEs and ACs. Table 8 shows the important economic results for the system. In the table, the TIC of the A-CAES + BMGES system is estimated as £2 374 370 representing a minimum A-CAES + BMGES system specific cost of about £2040. The estimated COE and TLCC for the system is 0.19 £ kW per h and £4.4 million respectively.

**Table 8:** Economic performance results for the integrated system

Parameter, (unit)	Value
TIC, (£)	2 374 370
NPV, (£)	−2 144 062
TLCC, ($)	4 421 655
COE, (£ per kW h)	0.19
Specific investment cost, A-CAES + BMGES (£ per kW_e_)	2040

**Specific cost, TEST (£ per kW**_**th**_ **):**	
TES_1_	24.32
TES_2_	25.81
TES_3_	17.53

**Shares of components in TIC (%):**	
*c*_AC_	15.41
*c*_AE_	13.45
*c*_HAD_	0.82
*c*_BMG+DFE,_	34.96
*c*_TES_	5.08
*c*_HX_	4.45
*c*_AS_	25.82

The total CAPEX value for thermal energy storage in the A-CAES + BMGES is estimated as £67.65 per kW h comprising of £24.31 per KW h for TES1, £25.81 per kW h for TES2 and 17.53 per kW h TES3 respectively. The estimated cost of TES values is within the range of £8.70–43.59 per kW h (€10–50 per kW h) reported as costs of latent heat storage systems based on PCMs.^82^ It can be observed that the specific cost of TES_1_ and TES_2_ (£ per kW h) are more than that of TES_3_ by over 40%. This is because, more heat is stored in TEST_3_ owing to higher HTF temperature entering it when compared with TEST1 and TEST_2_. In addition, the cold PCM temperature in TEST_2_ is higher than in TEST_3_ since the temperature of the compressed air from the air store entering TEST_3_ is at ambient. In TEST_1_ and TEST_2_, the cold temperature of the PCMs is set by the minimum TET from the AEs which is higher than ambient temperature. It is fascinating to assess the shares of cost of the different components of the A-CAES + BMGES system in the TIC as in Table 8. The CAES has the highest share of all the components of the system. It accounted for about 59.8% of the TIC consisting of 15.4% for AC, 13.5% for AE, 5.1% for TES and 25.8% for AS. The biomass gasifier with cleaning system and the DFE has the second highest share of 35% with the syngas cleaning system contributing 6.3%. The recuperator accounted for 4.45%. The NPV for the system is found to be negative with a value of £2 144 062 indicating the non-profitability of the system in the selected location.

### 4.1 Sensitivity analyses

#### 4.1.1 Impact of cost factors

**Fig. 5:**
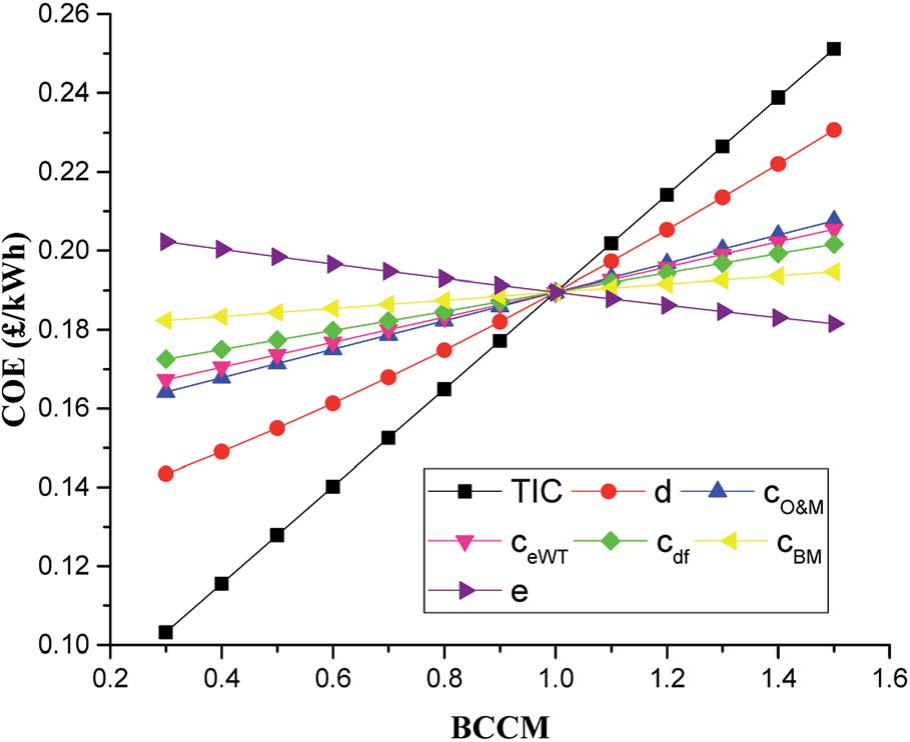
Impact of economic factors on COE.

The performance of the integrated system depends on a number of technical and cost factors. Cost factors include the total investment cost (TIC), the discount rate (*d*), the fuel price, the O&M cost (*c*_O&M_), inflation rate (*e*), and cost of excess electricity (sold and bought). The technical factors are the syngas fraction (sf), capacity factor (CF), round trip efficiency (RTE) of the A-CAES TES system, the syngas temperature (SGT) and the exhaust gas temperature (EGT) of the DFE. In the sensitivity analyses, the effect of changes in the technical and economic parameters were studied and analysed so as to evaluate their effects on the overall specifications of the system. Specifically, in the cost sensitivity, the base line value of each cost parameter input into the COE calculation is adjusted by multiplying each of the base line cost parameters by a base line cost component multiplier (BCCM) that ranged from a smallest value of 0.3 to highest value of 1.5 in accordance with IEA methodology for energy plants.^4^ Then a sensitivity plot (SP) of COE for the variation of the factors is illustrated. The greater the slope of the COE is with respect to the relative change in the cost factor, the bigger the influence on the COE and *vice versa.*Fig. 5 shows the results of this sensitivity analysis for the COE. As it can be observed, the TIC has the steepest slope of the factors and thus has the strongest influence on the COE. This is because TIC remain the single biggest element of the system's cost. If the TIC increases from its base line value by a BCCM of 1.3 (30% increase) while all other variables are held constant, the COE increases to 0.23 £ per kW h. This represents a percentage increase in the COE of about 19.5%. However, at a BCCM of 0.7 (30% reduction), the COE reduces by about 19.5% to 15.25p per kW h which is outside the range of current UK national average price for electricity which is 14.05p per kW h.^74^ This fairly high percentage change in COE with variation in the TIC highlights the significant sensitivity of the COE to change in the TIC.

The discount rate (*d*) has the second highest impact on COE. The COE is found to increase with increasing discount rate. This is because the discount rate influences the costs of capital. The higher the discount rate, the higher the cost of capital and *vice versa.* The COE for the discount rate varied from a low value of about 0.14 £ per kW h at a low BCCM of 0.3 (*d* = 3%) to a high value of about 0.23 £ per kW h at a BCCM of 1.5 (*d* = 15%), representing about 21.7% increase in COE from the base value.

The other parameters do not influence the COE significantly compared to the TIC and discount rate. Cost of O&M (*c*_O&M_) and excess wind electricity (*c*_eWT_) has third and fourth highest impact on the COE with COE increasing by about 9.5% and 8.4% in each over BCCM range of 1 (base value) to 1.5 respectively.

The COE is least sensitive to the cost of biomass (*c*_BM_), which sees only about 2.7% increase in COE as the BCCM is increased from base value to 1.5. The inflation rate (*e*) has the second least impact on the COE followed by the cost of diesel (*c*_df_). As can be observed the inflation rate (*e*) is inversely correlated to the COE, with higher *e* values resulting in lower COEs and *vice versa.* The COE increases by about 6.4% within the same BCCM range of 1 to 1.5 for cost of diesel fuel (*c*_df_).

**Fig. 6:**
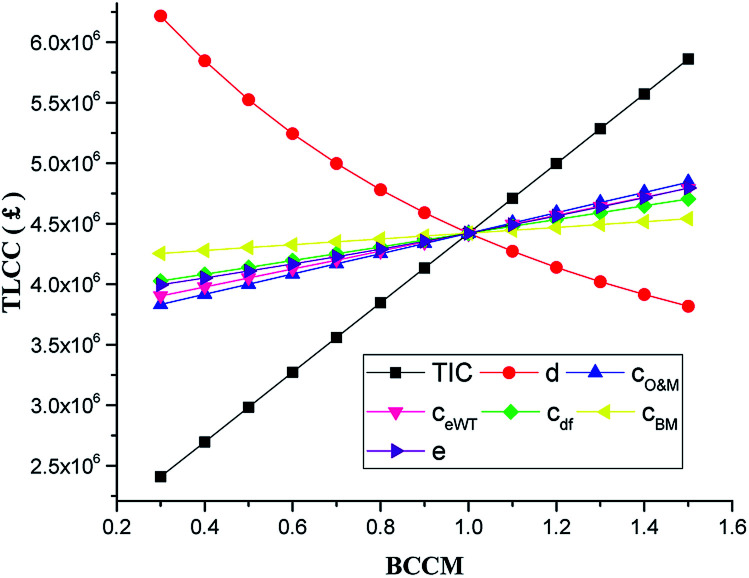
Impact of economic factors on TLCC.

The corresponding TLCC for the system under the factors accessed in the COE sensitivity above is shown in Fig. 6. The sensitivity of the factors to the TLCC also followed the same order as that in the COE with TIC impacting TLCC the highest and cost of biomass the least. One would have expected the cost of biomass to impact the system COE more since it is one of the fuel used by the system. However, because the system takes advantage of cheap waste heat to dry cheap wet biomass, the cost of biomass seemingly does not impact on the COE significantly. Along similar lines, the influence of changing the cost factors (TIC, *c*_BM_, *c*_eWT_, *c*_df_, *c*_O&M_, *c*_BM_) including the selling price for heat and electricity (*e*_tarrif_, *h*_tarrif_) on the NPV was analysed. The NPV is important because it is an indicator of how profitable the proposed system would be when implemented. The NPV of the system remained negative as the cost factors were reduced to BCCM of 0.4. This means the A-CAES + BMGES system is not profitable for heat and power generation in the analysed location. However, if 70% of TIC is provided for by means of a subsidy to the investor by the government as one of the ways to encourage RE uptake in the energy mix, the system become profitable with a positive NPV value of £132 474.9 and COE of 0.10 £ per kW h at the baseline discount rate.

#### 4.1.2 Impact of technical factors

**Fig. 7:**
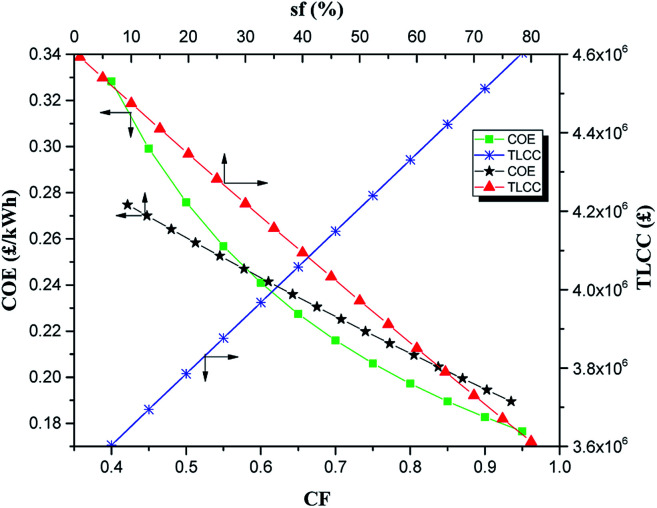
Impact of variation of sf and CF on COE and TLCC.

The syngas fraction (sf) of the DFE also plays a part in the overall efficiency and hence cost of the whole system. The sf was varied between 1% and 80% as in Fig. 7. As can be seen, the COE is highest at first and as the sf of the dual fuel engine decreases, the diesel fraction (DF) rises to keep the power output constant. The increase in the diesel fuel consumption leads to a corresponding increase in diesel cost (*c*_df_). Thus the effect of increase in diesel fuel cost (*c*_df_) at low sf resulted to a high COE of about 0.27 £ per kW h for the system at sf of 1%. This represents a COE increase of about 45% from the base value. As can be observed, the relative impact of the change of sf on the COE is significant for the system. The corresponding TLCC for the system is also shown in Fig. 7. Just like the COE, the TLCC is low at first when the sf is highest because of the reduced cost of fuel resulting from less diesel in the fuel mixture. As the sf decreases, the DF consumption and hence the cost of diesel (*c*_df_) increase with a consequent increase of the TLCC of the system.

The capacity factor (CF) of the integrated system also affects the COE. Just like the sf, the CF is also inversely correlated to COE, with higher CFs resulting in lower COEs and *vice versa.* This can be seen from Fig. 7. As the CF increases, the number of operating hours of the system increases resulting in the increase in generated energy by the system. On the other hand, as the CF reduces, the yearly operating hours of the system declines resulting in less power generation and increase of the COE of the integrated system. As can be seen in Fig. 7, the CF has a fairly strong effect on the performance of the integrated system. For a CF increase from 0.85 to 0.95, the COE reduces from 0.1895 to 0.1765 £ per kW h representing a decrease of about 7%. On the other hand, for a CF decline from 0.85 to 0.75, the COE increases by about 9%. Over the CF range of 0.4–0.95, the COE varied from a highest value of 0.328 £ per kW h to the lowest vale of 0.177 £ per kW h. The TLCC for the system has a linear relationship with CF because of increase in the number of operating hours and hence cost of O&M that resulted with increasing *CF.* It varied from a lowest value of about £3.60 million at CF of 0.4 to the highest value of £4.60 million at CF of 0.95.

**Fig. 8:**
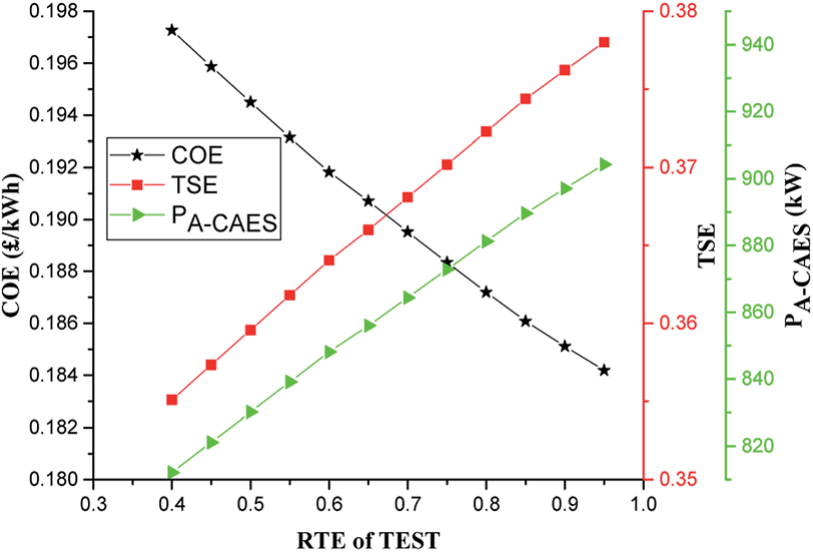
Impact of RTE of A-CAEs TEST on COE.

A less efficient TES system of A-CAES will generate less electricity than a more efficient storage system. The impact of RTE of the TES of the A-CAES on the COE, TSE and *P*_A-CAES_ of the system is shown in Fig. 8 over the range of 40 to 95%. As can be seen, the COE for the system varies inversely with the RTE. This is because, as the RTE of the A-CAES TES increases, the power generated by the A-CAES (*P*_A-CAES_) and hence the whole system increases as in Fig. 8, leading to the increase in the total system efficiency (TSE). The increased efficiency and power output of the A-CAES + BMGES at higher RTE thus leads to a reduced COE. A reduction of the RTE from the base value of 70% to 40% leads to about 4.1% increase in the COE to 0.1973 £ per kW h whereas an increase of RTE from the base value to 95% leads to about 2.8% reduction in the COE to 0.1842 £ per kW h. Over the RTE range of 40 to 95% the total system efficiency (TSE) increases from 0.355 to 0.38.

**Fig. 9:**
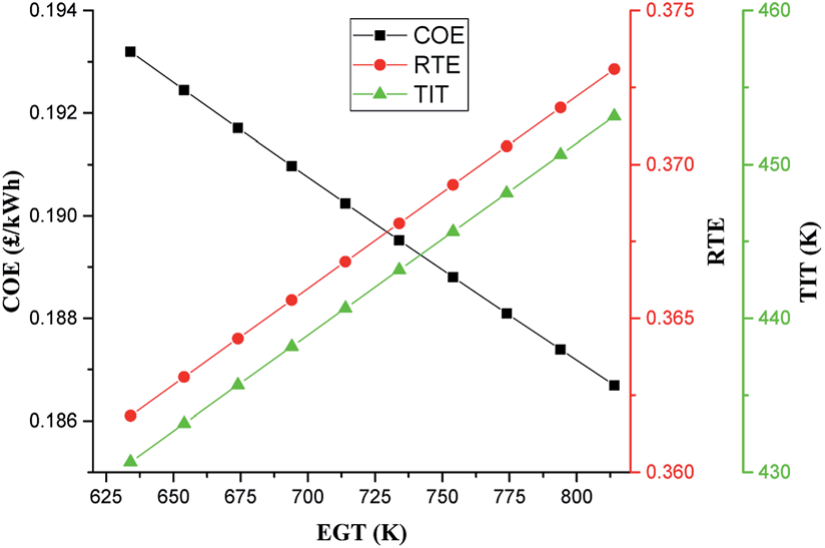
Impact of EGT on COE.

Fig. 9 shows the EGT sensitivity to the COE of the A-CAES + BMGES system. The most immediate and direct impact of EGT on the system is the variation of the TIT and hence TSE. In dual fuel mode, the EGT is usually higher than the diesel only mode at the same load.^83^ This may be due to slow combustion flame speed resulting from the incomplete combustion features of the syngas which leads to a fairly higher exergy loss through exhaust gas. The typical EGT of dual fuel compression ignition (CI) engine operating on diesel fuel and refined rice bran oil blends, having a compression ratio of 18.4 : 1 can vary from a low value of 341 °C at 63% load and 50% sf to a high value of 524 °C at 98% load and 75% sf, depending on the load, DF, compression ratio and ambient conditions.^84^

Based on 120 °C as the final stack temperature, varying the starting temperature of the DFE exhaust gas temperature from 361 °C (634 K) up to 541 °C (814 K) results in about 75% increase in temperature difference available and thus, available energy for recovery. The degree to which the energy obtainable in the higher EGT can be utilised depends on the characteristics of the system. Within the EGT range of studied, it is observed that increase in EGT improved the performance of the system. The TSE increased from 36.2 to 37.3%. The increase in the TSE is due to increase in available heat for recovery at high EGT which leads to increase in TIT as can be seen in Fig. 8. As expected the increase in the overall performance of the system at higher EGT led to improved COE of the system.

**Fig. 10:**
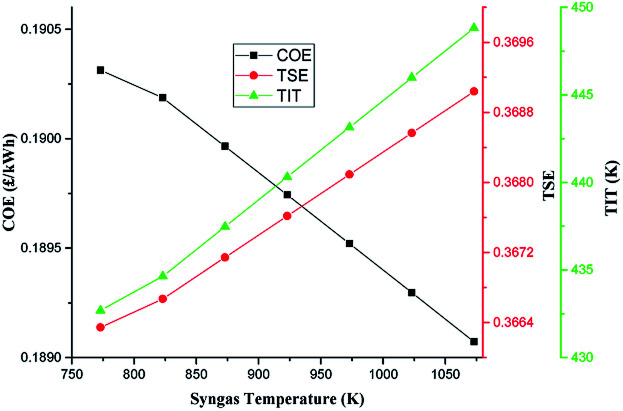
Impact of syngas temperature on COE.

Fig. 10 shows the sensitivity of COE of the system on the temperature of syngas (SGT) exiting the gasifier during the charging mode. As expected, the COE of the system varies inversely with the SGT. As the SGT reduces from the base case value of 973 K to 773 K, the COE increased from 0.1895 to 0.1903 £ per kW h. However, as the SGT is increased from the base value to 1073 K, the COE reduced to 0.1891 £ per kW h. The trend can be explained by the increase in the TSE with SGT. With increase in SGT from the base value to 1073 K, the higher TSE reveals the benefit of reheating on efficiency. TES efficiency rises with air re-heating since the increased turbine inlet temperature (TIT) in the AE stages leads to increased power outputs and hence TSE as can be seen in Fig. 9.

**Fig. 11:**
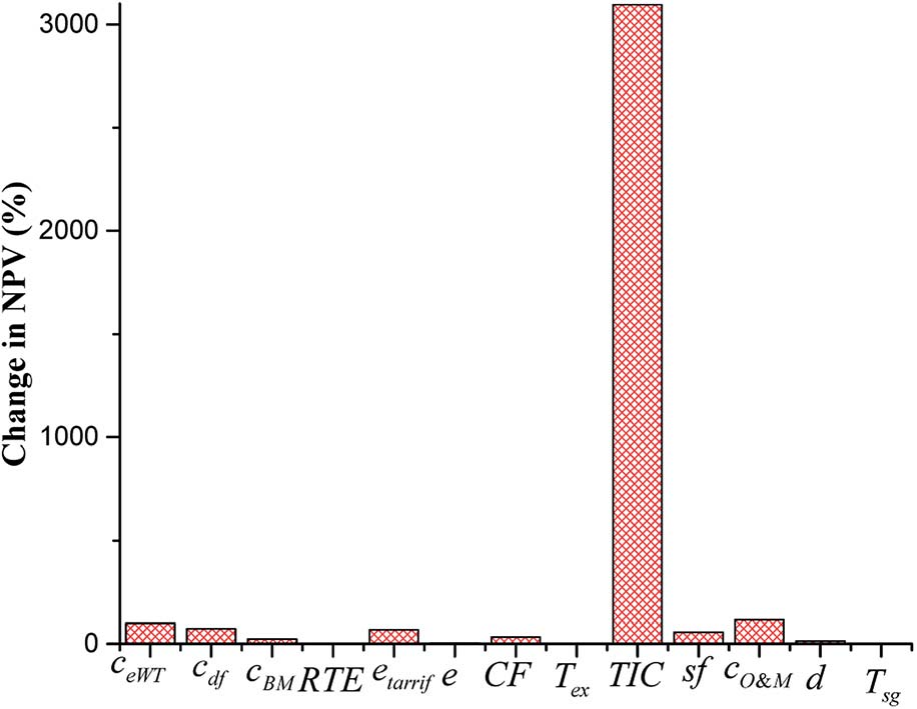
Variation of NPV with sensitivity cost parameters.

Fig. 11 shows the percentage variation of the NPV of the system to the variation of the sensitivity factors examined above from their minimum and maximum values. It can be seen clearly that the most significant factors swaying the NPV of the A-CAES + BMGES are TIC, cost of O&M, excess wind electricity cost, electricity tariff, and cost of diesel fuel

### 4.2 Limitations of the study

The result of the study should not be considered as representative of the UK or the world in any way. Electricity and heat demand including energy prices depends on a range of different supply and demand situations, including the geopolitical location, local climate, the national energy mix, network costs and taxation. Hence, the case study using Hull should only be considered as an example of the applicability of the methodology. The methodology can be scaled up to any capacity and used for any location or country while factoring in labour costs, fuels and electricity prices. Moreover, it should be noted the details of the hybrid systems cost and economics are valid elsewhere. The primary differences between the systems would be ensuring they meet the standards of the local regulators in the region of interest.

## 5. Conclusions and recommendations

A wind powered A-CAES system and a downdraft biomass gasifier with dryer coupled to a dual fuel engine are integrated in this paper to produce heating and power simultaneously for supplying some of the energy needs of 1600 households in Hull humber region.

Techno-economic analysis is carried out to investigate the performance of the system. The technical assessment focused mainly on electrical power generation, fuel consumption and efficiencies. The economic analysis is carried out based on total life cycle cost (TLCC), cost of electricity (COE), and net present value (NPV). Moreover a sensitivity analysis is carried out to appraise the effects of important technical and cost parameters on system performance. The main conclusions are summarized as follows:

• The results obtained indicates that the proposed system is technically possible for commercial distributed power and heat generation. It uses renewable energy and can simultaneously produce electrical and heating energy.

• Within the baseline design conditions, the system has the capability to produce a total electric power output of 1.16 MW (869.51 kW from A-CAES and 0.3 from DFE), 0.315 kW heat for sale and 318.03 kg of dry wood chips per hour with electric efficiency of about 29% and total system efficiency for the simultaneous production of heat and electricity of 36.8%. To achieve this, the system requires wind turbine excess electricity of 1 MW, a biomass gasifier with capacity to process 318.03 kg of wood per hour and a 0.3 MWe dual fuel engine with syngas and diesel consumption of 23.74 and 881.77 kg h^−1^ respectively.

• Results of economic analysis shows that the COE of the system (0.19 £ kW h) is outside the range of the average cost of electricity for a medium user home in the UK (2500 to 5000 kW h a year), including taxes which is 15.2p per kW h.

• Results of the sensitivity analysis of the technical parameters showed that increasing the EGT, RTE of A-CAES TEST and SGT increases the TSE of the system. In addition, it is concluded that since the A-CAES is interconnected with BMG and DFE, any variation in the parameter of BMG or DFE, will cause a variation in the performance of the A-CAES + BMGES system.

• The economic results of the system and the sensitivity (Fig. 5–10) provide investors with clear guides to the COE of the hybrid systems for distributed generation. The negative NPV of the system even with modest TIC reduction coupled with high COE cost as compared to global average COE of about 0.1 $ per kW h shows that the integrated system is not economically viable for distributed electricity generation in the selected location unless about 70% the TIC of the system is met by a government subsidy.

The economic position of the system may be further enhanced in the future by:

• Reducing CAES and BMG plant cost, which can occur through efficiency improvements as well as commercialisation.

• Increasing sell back to the grid tarrifs for power exported by private commercial generators.

• Granting high subsidy to investment in hybrid biomass and wind generation systems.

• Possible reductions in O&M, fuel, and other BOP costs have a moderately small effect, but of course should not be ignored as part of overall cost reductions.

It should be taken note of, that these results are independent of renewable energy certificates, feed-in tariffs or other government subsidies. The expected reduction in A-CAES and BMG costs due to commercialisation, standardisation, lower cost of capital and technology learning in the future is likely to improve the economy of the overall system.

## Conflicts of interest

The authors declare no conflict of interest.

## 6. Nomenclature

AC: Air compressor
AE: Air expander
AS: Air store
BMG: Biomass gasifier/gasification
BOP: Balance of plant
BY: Base year or current year
C: Cost ($)
Cc: Counter current
*c*_r,e_
: Rejected wind electricity cost
*c*_ect_
: Electricity consumer tariff ($ per kW h)
*c*_est_
: Electricity sell to grid tariff ($ per kW h)
*c*_ebt_
: Electricity buy back tariff ($ per kW h)
*c*_hct_
: Heat consumer tariff ($ per kW h)
*c*_hst_
: Heat sell back tariff ($ per kW h)
*c*_hbt_
: Heat buy back tariff ($ per kW h)
*c*_PCM_
: PCM cost
*c*_steel_
: Steel cost ($ per kg)
*c*_ins_
: Insulation cost ($ per m^2^)
cf_$–£_: Conversion factor of $ to £
cf_$−£_: Conversion factor of euro to £
CGE: Cold gas efficiency
*C*_p_
: Specific heat capacity (kJ kg^−1^ K^−1^)
CV: Calorific value (kJ kg^−1^)
CY: Currency year
*d*
: Discount factor (real)
*D*
: Annual Depreciation ($)
de: Deficit/imported electricity (kW)
*E*
: Power input (kW)
*h*
: Enthalpy (J kg^−1^)
HX: Heat exchanger
HV: Syngas heating value (MJ m^−3^)
hrs: Hours of operation in a year (hrs)
*L*_m_
: Specific melting enthalpy (J kg^−1^)
*ṁ*_a,D_
: Mass flow rate of air in dryer (kg s^−1^)
*ṁ*_D,AC_
: Mass flow rate of air in HX1 (kg s^−1^)
*ṁ*_D,sg_
: Mass flow rate of air in HX3 (kg s^−1^)
*ṁ*_BM,e_
: Mass dry feed at exit (kg s^−1^)
*ṁ*_BM,i_
: Mass of dry feed at inlet (kg s^−1^)
*ṁ*_w,i_
: Mass of water biomass at inlet (kg s^−1^)
*ṁ*_w,e_
: Mass of water biomass at exit (kg s^−1^)
*ṁ*_w,eV_
: Mass of water evaporated (kg s^−1^)
*n*
: Economic life (yrs)
*n*_s_
: Number of stages
*P*
: Pressure (bar)
PV: Present value
*Q̇*
: Flow rate (kg s^−1^, l s^−1^)
*Q*_TES_
: Thermal storage capacity (W)
*R*
: Gas constant (J kg^−1^ K^−1^)
*R*
: Revenues ($)
RH: Recovered heat (MJ)
*T*
: Temperature °C
Tax: Tax rate (%)
TLCC: Total life cycle cost ($)
Δ*T*_m_: log mean temperature difference (°C)
*V*
: Volume (m^3^)
*ṁ*
: Mass flow rate (kg s^−1^)
MC: Moisture content by wt (%)
*m*_r_
: *ṁ* ratio of the AE to the AC

## 7. Greek symbols

φ
: Humidity of air (kg kg^−1^)
ρ
: Density (kg m^−3^)
β
: Pressure ratio per compressor stage
γ
: Ratio of air specific heats
η
: Effectiveness/efficiency
ψ
: Specific fan power (kW m^−3^ s^−1^)

## 8. Subscripts

a: Air
B: Biomass/wood
BG: Biomass gasifier
c: Cold
ch: Charge/charging
D: Annual depreciation
d: Dry
dch: Discharge/discharging
df: Diesel fuel
e: Electrical/electricity
ev: Evaporation
ex: Exhaust
e: Final/leaving
h: Heat/hot
HA: Hot air
HX: Heat exchanger
i: Initial/entering
isen: Isentropic
insu: Insulation
M: Mechanical
Max: Maximum
min: Minimum
*n*
: Year
o: Out/outlet/overall
O&M: Operation and maintenance
r: Real
recup: Recuperation
ref: Reference
RH: Percentage relative humidity (%)
s: Sensible
T: Total
*t*
: Time (secs)
theo: Theoretical
vol: Volumetric
w: Wet/moisture
wb: Wet basis
wv: Water vapour
WT: Wind turbine

## Supplementary Material
